# Use of an upright power wheelchair in spinal cord injury: a case series

**DOI:** 10.3389/fresc.2024.1267608

**Published:** 2024-03-06

**Authors:** Eunkyoung Hong, Michael Elliott, Stephen Kornfeld, Ann M. Spungen

**Affiliations:** ^1^ Spinal Cord Damage Research Center, James J. Peters VA Medical Center, Bronx, NY, United States; ^2^ Department of Rehabilitation and Human Performance, Icahn School of Medicine at Mount Sinai, New York, NY, United States; ^3^ Department of Medicine, Icahn School of Medicine at Mount Sinai, New York, NY, United States

**Keywords:** standing power wheelchair, spinal cord injury, quality of life, usability, safety, tetraplegia, paraplegia

## Abstract

**Objective:**

To explore independence, usability, and self-reported quality of life (QOL) in eligible persons with spinal cord injury (SCI) who used a standing powered wheelchair over a 12-week period. Setting: VA SCI research facility.

**Participants:**

Four participants with chronic SCI who use a wheelchair as the primary means of mobility.

**Intervention:**

A standing power wheelchair was used three times a week (3.5 h/session) for 12 weeks in a supervised setting. Main Outcome Measures: safety, usability and feasibility, blood pressure in seated and standing positions, bowel, bladder, and pain item banks from the SCI-QOL Physical-Medical-Health domain, and overall user satisfaction with the device.

**Results:**

Participants consistently maintained normal blood pressure responses between seated and standing positions throughout the training sessions and learned to perform all the mobility tasks safely and independently. Participants reported improvements on the SCI-QOL and were generally satisfied with the upright standing power wheelchair.

**Conclusions:**

In this small case series of chronic, non-ambulatory individuals with SCI, the standing powered wheelchair was shown to be safe and efficacious.

## 1 Introduction

Approximately 27.2 percent of the US population has some type of disability and about 10 percent have a physical disability resulting in a mobility impairment. Approximately 18.4 million people use various assistive technology devices for mobility and 5.5 million people use a wheelchair (1, 2). Innovative wheelchair technology is integral for users to maintain a mobile lifestyle with enhanced function, increased independence, and greater accessibility in the home, work, and community. As a result, the wheelchair is the primary mobility device for this segment of society. As individuals using wheelchairs adapt to the use of a wheelchair in daily life, it soon becomes an extension of their bodies. Although wheelchairs provide mobility, people with spinal cord injury (SCI) who are non-ambulatory are at risk for many secondary medical consequences due to paralysis and the extreme amount of time spent sitting. The rate of wheelchair usage is increasing has led to a growing demand for better wheelchair solutions.

Reducing the time sitting has become a major goal to improve physical activity. Despite of ergonomic advanced office chairs, typically sitting for more than two hours has been associated with the development of pain (3, 4). As such, frequent standing and/or repositioning is recommended in an ambulatory population who sit for a prolonged period (5, 6). Ambulatory population studies correlate increased sitting with increased body mass index and mortality and reducing the amount of time sitting improves metabolic outcomes (7–9). Similarly, frequent wheelchair position changes are advised (5, 10, 11). While standing has benefits, current wheelchair solutions have limitations. A powered wheelchair (UPnRIDE) offers seated, stationary standing, and overground standing mobility. We tested the safety, useability, and user satisfaction on the UPnRIDE power wheelchair.

Our group has reported that exoskeletal-assisted walking has some positive effect on bowel function (12). Very little has been published on the effects of using a standing wheelchair. In one study by Dunn et al., they reported on usage of the device at one and five years; 84% of responders were using the standing wheelchair to stand, with 41% standing one to six times per week and that 21 of 99 surveyed reported improved bladder control, and a small unspecified number reported better bowel regularity, reduced urinary tract infections, reduced leg spasticity, and reduced bed sores (13). In contrast, Kwok et al., reported in a randomized controlled trial (RCT) that there was no effect from standing on time to first stool (14). We wanted to explore if long-term standing and frequent position changes would have a positive effect on bowel function and other on quality of life outcomes for those with SCI (15, 16).

The goal of this pilot project was to determine the effects of standing with the UPnRIDE powered wheelchair for extended periods of time in a supervised setting on safety, independence, usability, and QOL in eligible persons with SCI who typically spend most of their waking hours sated due to limited access to standing modalities.

## 2 Methods

### 2.1 Recruitment and screening

This study was approved by the Institutional Review Board (IRB) of the James J. Peters VA Medical Center (JJPVAMC), Bronx, NY and registered in the clinicaltrials.gov website listing (NCT04163796). The targeted study population was individuals with chronic SCI (≥6 months) who were non-ambulatory and therefore used a wheelchair for the primary mobility. The study SCI staff physician was the primary source for identifying potential participants. Additionally, IRB-approved flyers and brochures were distributed. Potential participants were informed about the details and eligibility for the study and given the opportunity to ask question before signing the informed consent. Consented participants were screened by a history and physical examination incorporating the following: the International Standards for Neurological Classification of SCI (ISNCSCI) examination to determine the level and completeness of injury; range of motion at the hips, knees and ankles bilaterally; and orthostatic tolerance test.

Patients with autonomic dysreflexia (AD) and/or frequent orthostatic hypotension (OH) are potentially those who may benefit the most from regular upright posture. These patients were not excluded because we have learned from our Exoskeleton Assisted Walking (EAW) studies that by titrating their time in a standing position, people with those conditions can adjust to tolerate upright posture with strict monitoring of blood pressure (BP) and symptoms. BP for adverse changes and clinical symptoms were frequently monitored during all sessions. If a systolic BP decrease of greater than or equal to 20 mmHg or a diastolic BP decreased of more than 10 mmHg occurred within 3 min of changing position and/or the participant was symptomatic, we immediately bought the individual back to sitting or a horizontal position. Additionally, if there was a trend towards and fall in BP or any mild symptoms presented, they were encouraged to return to a seated position. Any changes in BP were listed as expected risks in the protocol and in the consent form and did not warrant an Adverse Event report unless they remained unresolved with sitting or supine, which never occurred, because the BP reductions and symptoms all resolved with sitting and the participant went on to tolerate standing.

To rule out participants who may be at high risk for a fragility fracture from weight bearing during standing in the wheelchair, a bone mineral density (BMD) scan was performed on the bilateral knees (proximal tibia and distal femur) as well as the dual femur (femoral neck and trochanter) using Dual Energy x-ray Absorptiometry (DXA). In addition, individuals with other bone conditions indicative of a high risk of fracture were excluded at the discretion of the study physician's clinical judgement. The complete list of inclusion/exclusion criteria is described below (Table 1).

**Table 1:** Enrollment criteria.

Enrollment criteria
Inclusion criteria
1. Use a wheelchair as a primary means of mobility;
2. Males and females, between 18 and 65 years old;
3. Traumatic or non-traumatic tetraplegia or paraplegia >6 months in duration;
4. Height 160 cm–190 cm (63–75 in or 5’3”–6’3” ft);
5. Weight <100 kg (<220 lb);
6. Able to sign informed consent.
Exclusion criteria
1. Able to ambulate with or without an assistive device or physical assistance greater than 4 consecutive steps;
2. Any pressure ulcer at any location that is deemed to be contraindicated for a power wheelchair or standing frame by the study physician;
3. Concurrent medical disease that would be exclusionary for standing (as per the clinical judgment of the study physician);
4. Severe spasticity (Ashworth 4) or uncontrolled clonus;
5. History of fragility fractures, long bone fractures in the past 1 years, heterotrophic ossification, or other bone conditions that would be exclusionary for use of a standing modality as per the clinical judgement of the study physician;
6. Significant contractures that would be exclusionary for use of a standing modality as per the clinical judgement of the study physician;
7. Psychiatric or cognitive status that may interfere with the ability to follow instruction to use the device; and
8. Pregnant or lactating women.

### 2.2 Research design

An open-label, single group perspective pre- and post- intervention study was conducted using a convenience sample. The intervention consisted of approximately 3.5-hour sessions, 3 times per week over 12 weeks.

### 2.3 Device features and description

**Figure 1:**
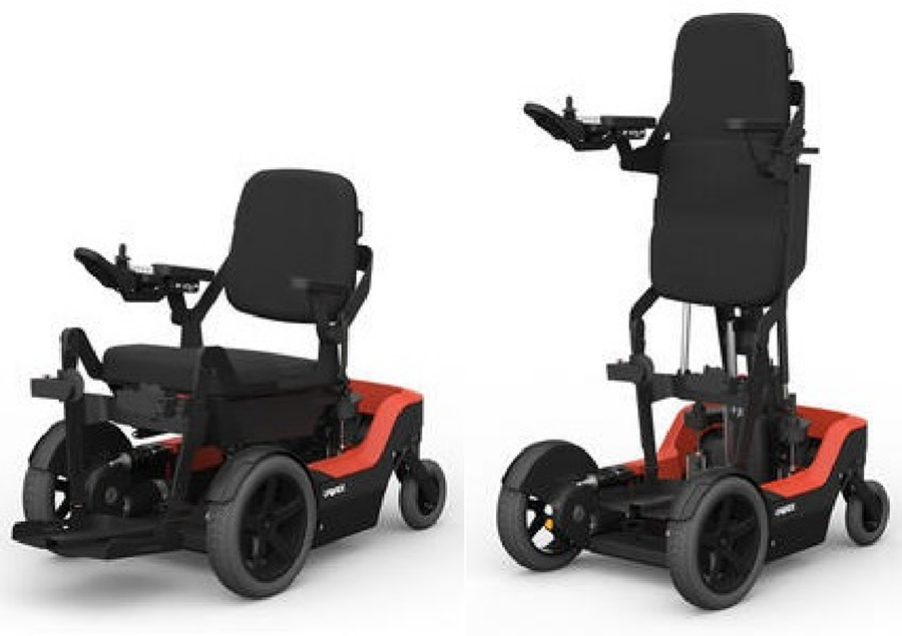
The UPnRIDE powered wheelchair. Image depicts the seated (Left) and standing position (Right) of the wheelchair.

The UPnRIDE powered wheelchair can change position from sitting to standing and standing to sitting or can be extended to a full supine position and can be used for indoor and outdoor mobility (Figure 1). The device is operated by the user with a joystick and a main computer controller and includes a driving motorized module, standing and sitting module, and stabilization module which can be adjusted and corrected to an upright position up to 7° while standing and 12° while sitting.

### 2.4 Training sessions

During the first session, the participants were fitted in the device and given instructions about transferring (with assistance when needed) in and out of the wheelchair, taught how to control the position functions, and the wheelchair mobility skills (17). Participants were asked to use the UPnRIDE three times per week for 12 weeks. Sessions lasted, on average 3.5 h, during which participants spent time in the UPnRIDE as well as the transfer in and out of the wheelchair. Participants were encouraged to use the wheelchair for 3–4 h per session, but times would vary based on when their transportation dropped them off or picked them up. During each 3.5-hour session, participants were asked to stand at least 5 min during every 15 min or more as tolerated to determine their tolerance level without causing any undue problems from standing, such as blood pooling in the lower extremities, lightheadedness, or other discomforts. The recommended standing time for the participants was based on their individual health status and level of injury, but mostly their self-reported tolerance to standing. Tolerance depended on a few factors including participant comfort and stability of their blood pressure. In the first few sessions adjustments were made to the wheelchair to increase user comfort. This included options such as changing the angle of the standing position, tightening, loosening, or swapping the straps for better comfort and supporting the user to the back of the chair. If blood pressure continued to decrease for a few minutes after standing or symptoms of orthostatic intolerance presented, the participant was returned to a sitting position. Typically, standing time was tolerated for a few minutes during the first few sessions and increased gradually over time.

Heart rate (HR), blood pressure (BP), total session time, time in standing position, count of sit-to-stand positioning changes, total distance of overground movement, and comfort scale (0 = N/A, 1 = Very Uncomfortable to 5 = Very Comfortable) were monitored during each session. Participants used the UPnRIDE on the hospital floors in the hallways and outside on the hospital grounds. The hospital grounds provided a variety of conditions that the participants could use the wheelchair such as ADA compliant ramps, curb cut-outs, side slopes, and grass (soft surface). In addition, the participants were encouraged to use the chair as they would in everyday circumstances. These tasks ranged from preparing food, reaching cabinets, transferring, playing card games, using a computer, and riding elevators. One subject took the UPnRIDE on a bus to a fast-food restaurant to pick up lunch. At all times during a session at least one member of the study staff was with the participants. This was a safety requirement by our IRB in the event of equipment malfunction. However, very little assistance was needed by the study team member during these activities. Vital signs were taken whenever a participant changed position. If a participant experienced any blood pressure instability, appropriate positioning was performed, and vital signs were monitored as frequently as once a minute.

### 2.5 Outcome measures

The primary outcomes were safety (number, relatedness, and severity of adverse events), usability, and feasibility determined from a variety of wheelchair mobility skills and an activities of daily living course. These were performed to assess an individual's functional independence. Blood pressure was measured in the seated and standing positions, and a spinal cord injury quality of life (SCI-QOL) measurement tool for bowel and bladder management difficulties, bladder complications, pain interference and pain behavior were used to determine whether reducing sitting time by a standing intervention had positive changes on these variables. The measurements were performed 3 times: pre- (baseline) and post-testing (after 36 sessions). Following completion of the study, each participant was asked to complete a questionnaire of the overall satisfaction of using the UPnRIDE.

#### 2.5.1 Safety with blood pressure during seated and standing positions

Blood pressure and heart rate (HR) were measure by a vitals monitor (GE Medical CARESCAPE V100 monitor) (18) and were monitored frequently during seated and standing positions for every session.

#### 2.5.2 Usability and feasibility

A modified Wheelchair Skills Test (WST) was used to assess the difficulty level of participants in completing mobility skills while using the UPnRIDE. The grading for the WST for mobility skills with the UPnRIDE was as follows: 0 = fail, 1 = pass with difficulty, and 2 = pass. The original WST is a comprehensive and generic instrument for objectively evaluating wheelchair skills (19). However, since the UPnRIDE is a standing power wheelchair, additional operations of sit-to-stand and stand-to-sit were included in the modified WST.

#### 2.5.3 Quality of life outcomes

The SCI-QOL measurement tools for the physical-medical health domain for bowel and bladder management difficulties, bladder complications, pain interference and pain behavior were performed at the three study time points. Lower scores indicate more positive responses, and a five-point decrease is considered to be a clinically meaningful improvement (20, 21). The SCI-QOL Bowel Management Difficulties SF9a and Bladder Management Difficulties SF8a scales use the following response options: “Not at All (1),” “A Little Bit (2),” “Somewhat (3),” “Quite a Bit (4),” and “Very Much (5).” Meanwhile, the SCI-QOL Bowel Management Complications scale has six questions with response options of “Never (1),” “Rarely (2),” “Sometimes (3),” “Often (4),” and “Always (5).” Regarding Pain, the Pain Behavior scale had 3 questions that were scored as follows: “Never (1)”, “Rarely (2)”, “Sometimes (3)”, “Often (4)”, “Always (5)” and 4 questions that were scored as follows: “Had No Pain (1)”, “Never (2)”, “Rarely (3)”, “Sometimes (4)”, “Often (5)”, “Always (6)”. The Pain Interference Short Form had 7 items that were scored as follows: “Not at All (1)”, “A Little Bit (2)”, “Somewhat (3)”, “Quite a Bit (4)”, “Very Much (5)” and 3 questions that were scored as follows: “Never (1)”, “Rarely (2)”, “Sometimes (3)”, “Often (4)”, “Always (5)”.

#### 2.5.4 Overall satisfaction

An Overall Satisfaction questionnaire was designed to measure participants' reactions to using the UPnRIDE. The questionnaire used a 5-point Likert scale (1 = Very Poor, 2 = Poor, 3 = Moderate, 4 = Good, 5 = Very Good). Participants were asked to rate their ability to adjust the device's position when performing certain activities. Ratings greater than three are favorable responses. Participants were also asked what they liked most and least about the UPnRIDE wheelchair based on their experience using an open response.

### 2.6 Data analysis

Since the sample size of this pilot study was small (*N* = 4), each participant's data is reported as a case series. The continuous variables were reported in mean plus or minus standard deviation (SD) for each individual. Total standing time over 36 sessions and the average number of times changing position were calculated and reported as mean ± SD to determine each of the participants' overall performance during this study.

## 3 Results

### 3.1 Participants

A total of seven participants were enrolled between 15 November 2018 and 13 March 2020. Four participants had completed 36 sessions when in-person research visits were not permitted due to the COVID-19 pandemic. After 18 months of study closure, loss of funding did not permit the re-start of the study. Therefore, we are reporting on the four participants who completed the 36 sessions as a case series. Any missed sessions (due to weather, transportation, etc.) were added on to the length of the training period, when possible, to achieve a total of 36 sessions The average length of the training period took three to four months to complete the total sessions. Demographic information for gender, height, weight, duration of injury, level of injury, and ISNCSCI classification are listed (Table 2).

**Table 2:** Demographic information.

ID	P1	P2	P3	P4
Age (Years)	54	56	45	41
Height (m)	1.7	1.72	1.63	1.75
Weight (kg)	89	74	100	79
BMI (kg/m^2^)	26.15	21.51	30.67	22.68
Sex	Male	Male	Female	Male
Duration of injury (Years)	31	31	3	5
LOI - AIS classification	C5 - AIS A	C4 - AIS D	T4 - AIS C	T6 - AIS A
UEMS right	16	22	25	25
UEMS left	14	25	25	25
LEMS right	0	8	1	0
LEMS left	0	8	0	0

P1-P4, Participant and number; M, meters; KG, kilograms; LOI, level of injury; AIS, American spinal injury association impairment scale; UEMS, upper extremity motor score; LEMS, lower extremity motor score.

### 3.2 Safety

There were no study-related serious adverse events (SAE) or adverse events (AE) that occurred during the use of the device or while the four participants were enrolled in the study. The four participants had appropriate HR and BP responses throughout the training sessions. HR and BP are two crucial physiological parameters that can be affected by changes in position, particularly in those with SCI who may experience sudden falls in BP, it is notable that none of the participants experienced a decrease of at least 20 mmHg in systolic blood pressure or a decreased of at least 10 mmHg in diastolic blood pressure within 3 min of changing from a supine to a sitting position (22). If a participant's blood pressure had a decrease or they had symptoms, they were encouraged to return to a seated position. These changes in BP were listed as expected risks in the protocol and consent form and did not warrant an Adverse Event report unless they remained unresolved with sitting, which never occurred, because the BP reductions and symptoms resolved with sitting and the participant went on to tolerate standing.

There were no HR or BP-related AEs while using the UPnRIDE, though in the early sessions 2 of the participants had lower BP and were brought to a seated position as a precaution.

**Figure 2:**
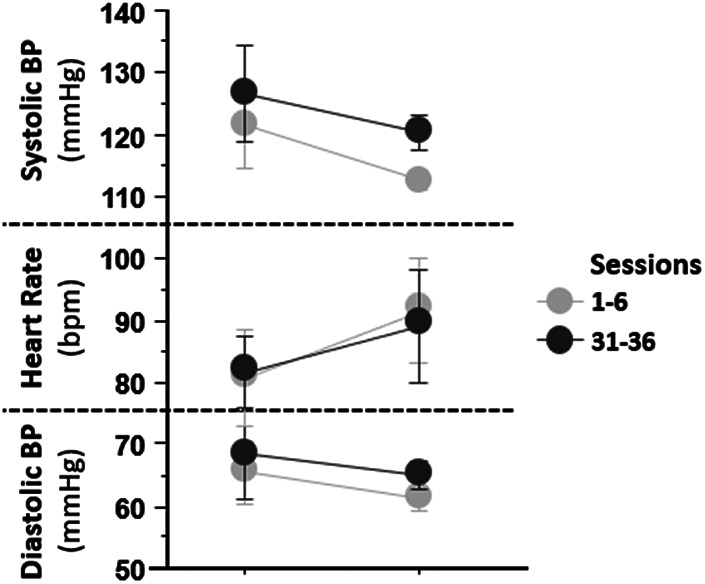
Hemodynamic results by session block from the first (1–6) and last 6 (31–36) sessions. Average systolic and diastolic blood pressures and heart rates are reported on the *y*-axis. On the *x*-axis are session block. The graph presents decreasing diastolic BP and systolic BP, increasing heart rate during seated and standing positions from the first to the last, and BP was well tolerated over all sessions.

Overall, participants had appropriate HR and BP responses throughout the training sessions (Figure 2). Systolic and diastolic blood pressures decreased with standing. HR increased with standing, all within the expected range.

### 3.3 Usability and feasibility

Participants learned to independently perform the mobility tasks of sit-to-stand and stand-to-sit, over smooth and various ground surfaces while in the upright standing position and to navigate the activities of daily living course (ADLC). The individual skills to operate the battery charger and engage/disengage the motors require fine motor skills of the upper extremities. As such, participants with limited hand function had difficulty operating the battery charger and engaging and disengaging the motors. Therefore, research trainers assisted operation of the charging cable. However, controlling a joystick and pushing buttons to adjust positions was possible using the wrists. The participant-reported comfort scales with performing mobility skills during training were 4 = Comfortable or 5 = Very Comfortable. During the 3.5-hour sessions over 12 weeks, all four participants were able tolerate more standing time than siting time. Because of the design of the device, the leg rests created a barrier between wheelchairs, and participants found it challenging to transfer in and out of the UPnRIDE wheelchair. Transferring required effort and time for the development of specific strategies (Table 3).

**Table 3:** Usage of UPnRIDE wheelchair.

ID	P1	P2	P3	P4
Average standing time per session (minutes)	95.6	89.8	90.2	147.9
Average sitting time per session (minutes)	63.7	75.3	22.6	27
Completion time (days)	126	105	130	105

P1-P4, participant and number. P1 is missing standing and sitting times from a datalogger malfunction. However, the times were estimated from session start/end time, since P1 was asked to change positions every 15 min. The average duration that participants spent using the device was 182 min. The minimum amount of time recorded was 67 min, while the maximum duration was 324 min.

### 3.4 Bowel, bladder and pain item banks from the physical-medical-health domain of the SCI-QOL

**Figure 3:**
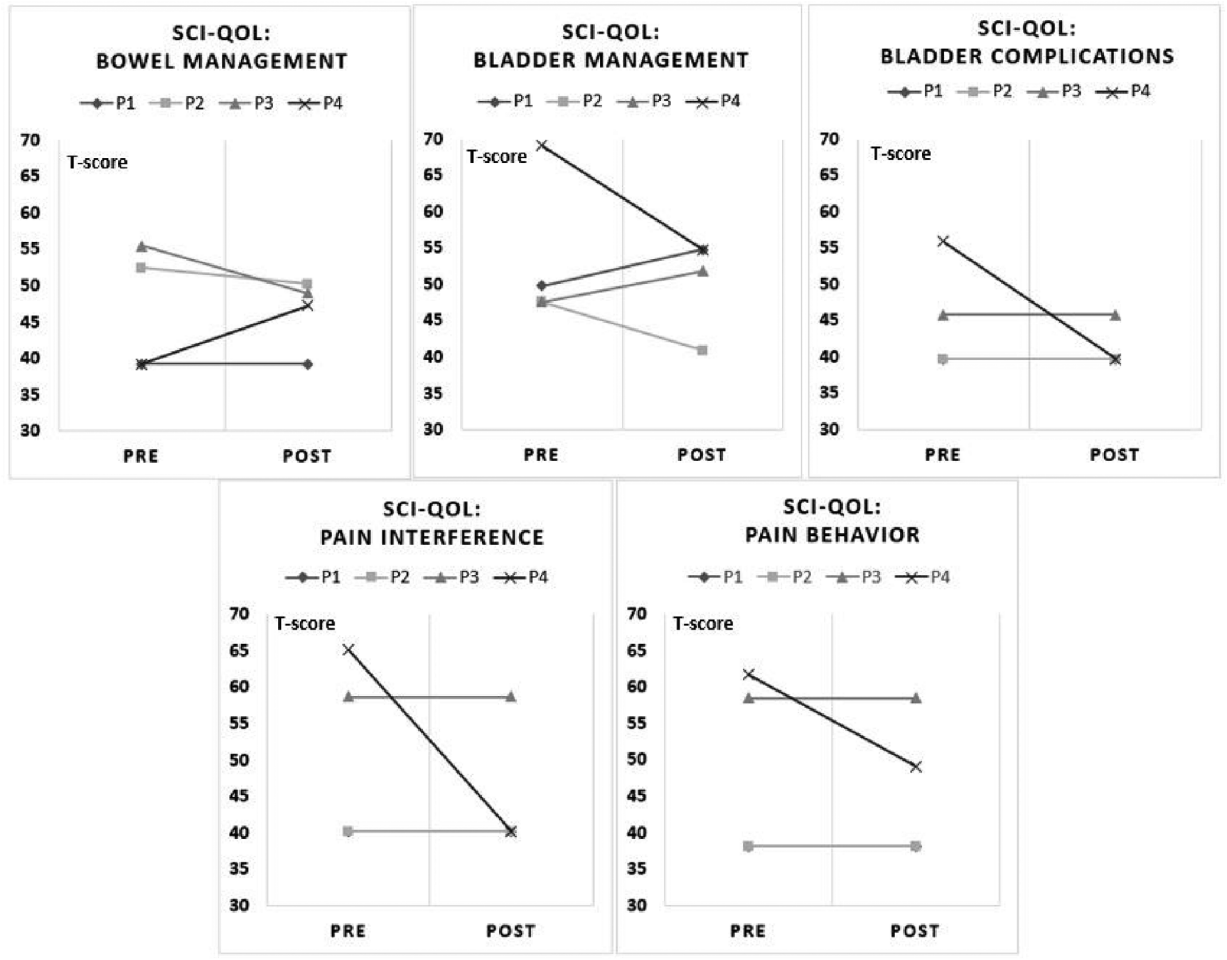
Charts of pre-post SCI-QOL by categories. Each panel depicts five components of the SCI-QOL physical-medical-health domain (18, 19). A reduction in scores from pre to post indicate an improvement.

The results from the SCI-QOL Physical-Medical Health domain for bowel and bladder management difficulties, bladder complications, pain behavior, and pain interference are reported (Figure 3). P1 reported bladder management difficulties were complicated after using UPnRIDE. Other categories stayed the same before and after using UPnRIDE for this individual. P2 reported improvements on bowel and bladder management, but the other outcomes stayed the same. P3 reported improvement on bowel management but had more difficulties with bladder management. Lastly, P4 reported worsening bowel management but reported improvements on bladder management and reduced bladder complications, pain interference, and pain behavior.

### 3.5 Overall satisfaction

Participants were generally satisfied with the UPnRIDE wheelchair (Mode = 5: Very Good), receiving positive responses for support, stability, reclined position while resting, and stability of standing position. The ratings from participants for ease of transferring, stability of adjusting, and comfort of adjusting were moderate. The individual specific feedback for overall satisfaction is reported (Table 4).

**Table 4:** Participant-reported overall satisfaction with UPnRIDE standing wheelchair.

	P1	P2	P3	P4
Comfort	4	4	4	5
Feel of the ride	4	4	5	5
Support	5	4	5	5
Stability	5	4	5	5
Ease of transferring	3	3	4	5
Body position of adjustment	4	3	4	3
Stability of adjusting	3	3	4	3
Comfort of adjusting	3	3	4	4
Reclined position while resting	5	4	4	5
Stability of standing position	5	4	5	5
Comfort of standing position	4	4	5	5
Usefulness with daily life	4	4	5	5
Pressure relief	5	4	5	5
Things liked MOST	(1) Ability to operate outside while standing. (2) Pressure relief of sore while standing and continue daily activity.	(1) The ability to stand. (That would come in handy at work.)	(1) The ability to stand at eye level.	(1) The ability to stand. (2) Weight on legs and Pressure relief of butt.
Things liked LEAST	(1) Difficulty transferring. (2) Tight Upper straps.	(1) Battery power goes quickly (3–4 h with frequent changes of position), needs extra batteries.	Nothing reported	(1) Not liking the position change from seated to standing felt awkard and like they were leaning too far forward.

Rating scale: 1 = Very Poor, 2 = Poor, 3 = Moderate, 4 = Good, 5 = Very Good.

## 4 Discussion

In this pilot safety, feasibility, and useability study, it was demonstrated that the four participants could independently perform the mobility tasks of sit-to-stand and stand-to-sit as well as maneuver the wheelchair over multiple ground surfaces while in the upright standing position. The participants were able to spend more time standing than sitting. The participants reported overall satisfaction of “good” with the device. Using a standing power wheelchair three times per week for 12 weeks was beneficial to increase BP tolerance with changing position. Results from the BP between seated position and standing position showed all participants were able to tolerate changing positions. Since there is no consistently effective single treatment for orthostatic hypotension (OH) in SCI, combining and individualizing management could provide OH tolerance (23–25). A couple of practical nonpharmacologic treatments to minimize hypotensive effects such as adjusting activity time and position adjustment were done with using the UPnRIDE. Therefore, using the standing power wheelchair was beneficial for a progressive tolerance to upright posture.

A passive position change may not be as beneficial as physical activity. In the participants tested, all were unable to conduct an active position change from sit to stand. Our study focused on safety, tolerability, and the participant satisfaction with the device. A future controlled and appropriately powered study to determine the effect of regular passive standing would be indicated.

For the category, “Pressure Relief”, participants rated the UPnRIDE as “very good”. Especially P1, who had a skin issue which needed frequent pressure relief of an area. P1 reported a benefit from using the position changes of the UPnRIDE standing power wheelchair and the participant was very satisfied with the pressure relief. Since the UPnRIDE standing wheelchair provides various seated functions, participants were satisfied with the reclined position while resting and the stability of standing position.

In the current version of the UPnRIDE device limitations with transferring were reported because of the static knee hinges. Although participants were satisfied with the UPnRIDE overall, it was difficult for them to independently transfer in and out of the device. Participants reported being uncomfortable with the stability of the device while adjusting positions but felt secure once in the standing position.

Patient-reported outcomes for bowel, bladder and pain were variable. P2 (AIS D) had two bowel accidents during the middle sessions, then did not have any accidents for the remainder of the of study sessions, likely attributable to subsequently requiring the participant to empty their bowel and bladder on the days prior to their sessions. The act of standing did not appear to have any appreciable benefits for pain reduction, particularly in the case of P3. P3 had chronic pain with and without using UPnRIDE. P4 had improvements on most categories, except bowel management. P4 is a manual wheelchair user, so the participant reported being able to use his upper extremities more freely with a greater range using the UPnRIDE. However, the extra safety security systems (chest harness, knee brackets, and seat belt) posed difficulties with doffing the device when needing to managing bowel movements compared with using a manual wheelchair with one seatbelt. Because of the variability among the four participants for the patient-reported outcomes on bowel, bladder, and pain, more participants need to be tested to draw any conclusions.

## 5 Limitations

A major limitation of the current case series is that the study was conducted with a small sample size with different levels and completeness of SCI. The training duration for each session varied and was primarily based on the availability and tolerability of the participants. Therefore, these findings are not generalizable. Knowledge from this data may serve as a basis for other clinical studies to establish standing protocols in other existing standing wheelchairs or ones yet to be developed.

The design of the device's leg rests presented limitations for participants when transferring in and out of the UPnRIDE wheelchair which required assistance and significant effort and time. Three more subjects were enrolled in the study at the time a research hold was placed due to the Covid-19 pandemic and were unable to complete the study.

## 6 Conclusions

This upright, standing wheelchair provided users a new level of mobility and freedom of upper extremities. Using the UPnRIDE also required less upper body function than current exoskeletons making it more practical for a wider range of people with SCI. The standing position supports stretching of the lower extremities. This is an important consideration because contractures restrict not only standing and walking, but dressing and other activities of daily living. Maintaining range of motion in the lower extremities would be needed for participation in further technological advancements that use upright positions.

The current case series in four participants suggests that use of this power wheelchair is feasible for upright overground mobility. Using an upright standing power wheelchair was demonstrated to be safe, feasible, and effective within one session of training. Most participants performed all functions of mobility skills and reported “comfortable/very comfortable” on the comfort scales. Appropriate HR and BP responses were demonstrated throughout the training sessions and the BP difference from seated to standing position decreased by the end of study. Some participants reported reductions in bladder complication, pain interference and pain behavior. Also, they were generally satisfied with the device, especially with support, stability, reclined position while resting, and stability of standing position. There may be greater benefits if participants are able to use the device for a longer period of time, such as during home use. Various upright wheelchairs, such as Permobil, Quickie, and Ki Mobility, offer distinct features and capabilities. Though not as mobile as exoskeletons or the UPnRIDE, they may still provide advantages to individuals with SCI who cannot use exoskeletons.

## Data Availability

The raw data supporting the conclusions of this article will be made available by the authors, without undue reservation.
